# Single-shot off-axis digital holographic system with extended field-of-view by using multiplexing method

**DOI:** 10.1038/s41598-022-20458-3

**Published:** 2022-09-30

**Authors:** Manoj Kumar, Lavlesh Pensia, Raj Kumar

**Affiliations:** 1 CSIR-Central Scientific Instruments Organisation, Sector 30C, Chandigarh, 160030 India; 2 Academy of Scientific and Innovative Research (AcSIR), Ghaziabad, 201002 India

**Keywords:** Engineering, Optics and photonics

## Abstract

We propose a new configuration of single-shot off-axis digital holographic system to realize double the camera field-of-view (FOV) of the existing off-axis Mech-Zehnder type holographic setup. The double FOV is obtained by double spatial frequency multiplexing of two different areas of an object beam by inserting a Fresnel bi-prism in it, which divides the object beam into two, both carrying different object information. The image sensor is placed at the plane where these two different FOVs overlap so as to record simultaneously two parts of the wavefront of the object in a single-shot. The multiplexed hologram is carrying two interferometric images corresponding to two different FOVs of the object which are modulated with two different spatial carrier frequencies. The feasibility of the proposed digital holographic system is experimentally demonstrated by imaging two different areas of a resolution test target. The limitation of the proposed system and a method to overcome it, are also discussed. The proposed system is useful in a wide range of applications including microscopy and optical metrology.

## Introduction

Digital holography (DH) is one of the most versatile multidimensional imaging techniques that use electronic devices to record and numerically retrieve the complex-amplitude distribution of an object wavefront^1^. DH has significant applications in various fields including static and dynamic deformation and displacement measurement^2–4^, biomedical imaging and microscopy^5–8^, encryption^9^, object recognition^10^, display^11^, and information storage^12^, because it allows fast, non-destructive, and full-field measurement of the specimen. Over time, DH has significantly evolved because of substantial developments in digital recording and numerical image processing technologies. However, DH has still several limitations such as limited field-of-view (FOV), which hinders its application in developing commercial systems based on this technology and there is an urgent need to develop efficient methods for FOV extension in DH. The FOV and resolution are the vital parameters of any imaging system as their ratio determines the space-bandwidth product (SBP = FOV/Resolution) of the system. The SBP is a measure for the information capacity an optical system possesses and it should be higher in order to acquire more object information and makes the measurement richer. In comparison to optical holography, DH systems have restricted SBP and resolution of reconstructed images because of the limited pixel pitch/size and pixel number, and low cutoff frequency of the image sensor (CCD/CMOS), which is much smaller than the observable FOV by a lens. There is a trade-off between resolution and FOV, so it is difficult to obtain a digital holographic image with high resolution and a wide FOV at the same time.

Several effective approaches have been proposed to overcome this limitation since its invention. The simplest method to extend the FOV is to increase the pixel number of the recorded digital hologram, however, commercially available image sensors currently have limited pixel numbers. Another method is the synthetic aperture technique^13^, in which a hologram is composed of bigger size from several recorded holograms under different conditions^14–18^. In this method, several holograms need to be recorded, therefore, requiring high stability and complicated calibration of the system. The FOV can be increased by recording multiple holograms of different areas of the object by mechanically translating the object and/or image sensor. However, such an approach has various limitations such as the entire process being time-consuming, the imaging speed is very low, and it is inappropriate for monitoring dynamic events. In recent years, the multiplexing approaches with different geometries and the use of special optical components^19–23^, to record different areas of the object in a single-shot, have been developed and proved to be more efficient ways to extend the FOV of the imaging system.

In this work, we present a new method of optically multiplexing two FOVs into a single off-axis interferogram, by using a special optical component: the Fresnel bi-prism, in the object beam, to double the FOV in DH. Several researchers have employed either special optical components (including the grating, Fresnel bi-prism, Wollaston prism, Rochon prism, beamsplitter, beam displacer, etc.) or optical configurations in DH to generate the object and reference beams needed for making the interference pattern between these beams^24–33^. In Ref.^24^, a lens-less cyclic lateral shearing DH for quantitative phase contrast imaging is proposed. The optical configuration is a self-reference type in which the object and reference beams are generated from the same spherical wavefront after the light beam has passed through the sample plane. The optical configuration is implemented with a beam splitter and mirrors. Due to the overlapping of the two wavefronts, the interference is formed in a small region (a region smaller compared to the CCD/CMOS sensor area), therefore, effective FOV is smaller than the sensor FOV. Sun et al.^25^ demonstrated interferometric microscopy that uses the combination of a cube beamsplitter and a Fresnel bimirror. The tilted beamsplitter divides the incident beam into two replicas (with π phase shift) and the Fresnel bimirror is then employed to interfere these two replicas with each other at the image plane of the image sensor. Therefore, two interference channels are acquired with a relative π (rad) phase shift in one interferogram, resulting in a lower FOV. A common-path phase imaging system is reported^26^ that uses a grating to divide the object beam into 0 and ± 1 orders. The 0th order is spatially filtered to erase all object information to make a clean reference beam which is allowed to interfere with the + 1 order (the object beam) to form the interference pattern. The FOV is equivalent to the sensor FOV in this configuration. In Ref.^27^, the authors have demonstrated the use of a beam displacer in a combination of polarizers in the path of the input beam to introduce a small displacement in two orthogonal polarized beams with a small displacement. These beams interfere after passing through another polarizer and form interference at the lateral shearing region behind the beam displacer. On the contrary, the optical configurations proposed by Lee and Park^28^ and Kim et al.^29^ use Wollaston and Rochon prisms respectively, to generate two orthogonal polarization beams and the interference occurs after passing through the polarizer placed before the image sensor. A pair of GRIN lenses, in the path of the collimated object beam, is used which yields a pair of high numerical aperture focal spots in their common output plane and the interference between these output beams fills the camera array^30^. Fresnel bi-prism has found its applications in several optical systems, e.g. for making common-path optical configurations^31,32^. In these systems^31,32^, the Fresnel bi-prism is employed to divide the incident object beam into two beams where one serves as the object beam and another reference beam, and finally, these two beams are allowed to interfere to form the digital hologram. The Fresnel bi-prism is also used in the digital holographic system to enable high temporal sensitivity, stability, high speed, high accuracy, and high spatial resolution^33^. The research work demonstrated in Refs.^23–33^ is the case of limited FOV where the interference region is smaller in comparison to the sensor FOV. Contrarily, the optical configuration, we proposed in this work utilizing the Fresnel bi-prism to extend the FOV, is different from the optical configurations presented in these works.

**Figure 1:**
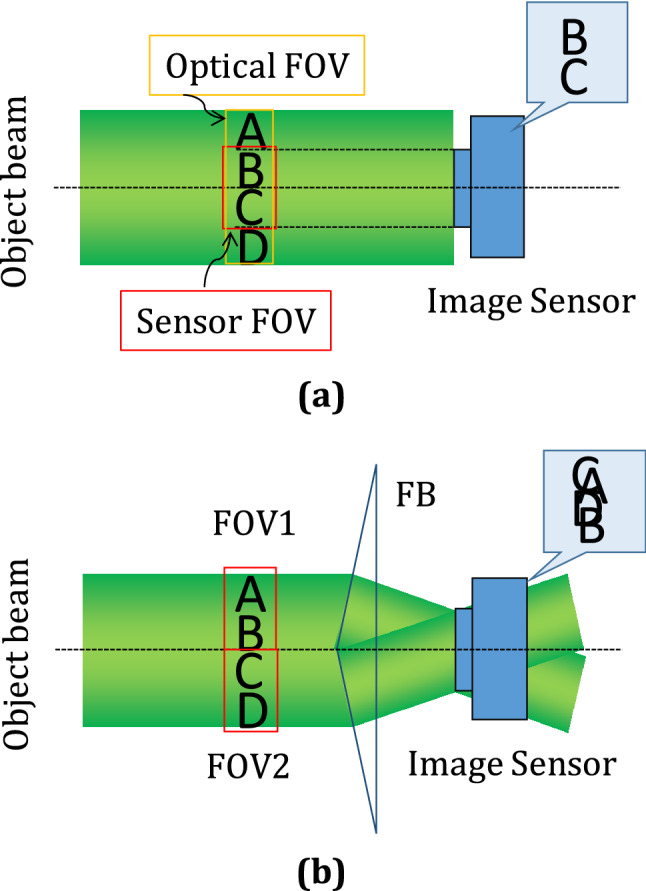
Two cases of FOV: (**a**) Limited FOV: without the use of Fresnel bi-prism records only the sensor FOV, and (**b**) Double FOV: with the use of Fresnel bi-prism to record two FOVs simultaneously.

The Fresnel bi-prism creates two FOVs when placed in the collimated object beam. The image sensor is placed at a plane where two different FOVs overlap, as shown in Fig. 1b. Therefore, two FOVs can simultaneously be recorded in single-shot. A plane reference beam, acting as a common reference beam for both the FOVs, is added to form a multiplexed digital hologram. Figure 1a depicts that the image sensor captures only a part of the object wave, termed sensor FOV, due to the limited active region of the sensor. On the other hand, Fig. 1b shows the proposed geometry, employing the Fresnel bi-prism, by which the same active region of the sensor can simultaneously capture two FOVs with different object information, in a single acquisition. Therefore, the present setup enables recording more interferometric information into a single multiplexed hologram in comparison to an optical holographic system.

## Theory

The digital holograms are generated by coherent mixing of the object beam $E_{o} (x,y)$ and reference beam $E_{r} (x,y)$, and recorded by an image sensor (e.g., charged-coupled device (CCD) and complementary metal oxide semiconductor (CMOS)). The object beam and reference beam can be represented as^34^

1 $$E_{o} (x,y) = A_{o} (x,y)\exp ( - j\phi_{o} (x,y))$$

2 $${E_{r} (x,y) = A_{r} (x,y)\exp ( - j\phi_{r} (x,y))}$$

where *A*_*o*_(*x*, *y*) and *ϕ*_*o*_(*x*, *y*) represent amplitude and phase distributions, respectively, of the object beam, and *A*_*r*_(*x*, *y*) and *ϕ*_*r*_(*x*, *y*) represent amplitude and phase distribution of the reference beam, respectively, and *j* = √−1. The intensity distribution of interference patterns *E*_*h*_(x, y) obtained at the hologram plane after coherent mixing of the object beam and reference beam, can be represented as,

3 $$E_{h} (x,y) = \left\{ {E_{o} (x,y) + E_{r} (x,y)} \right\}\left\{ {E_{o} (x,y) + E_{r} (x,y)} \right\}^{*}$$

4 $$\begin{gathered} E_{h} (x,y) = \left| {E_{o} (x,y)} \right|^{2} + \left| {E_{r} (x,y)} \right|^{2} + E_{o} (x,y)E_{r}^{*} (x,y) \hfill \\ \quad\quad\quad\quad\quad+ E_{o}^{*} (x,y)E_{r} (x,y) \hfill \\ \end{gathered}$$

In Eq. (4), first two terms $\left| {E_{o} (x,y)} \right|^{2} + \left| {E_{r} (x,y)} \right|^{2}$ on the right-hand side are the constant terms of intensity, $E_{o} (x,y)E_{r}^{*} (x,y)$ and $E_{o}^{*} (x,y)E_{r} (x,y)$ represent the complex amplitude of the object beam and its complex conjugate respectively. This intensity distribution, digitized by the image sensor and recorded as a digital hologram, is stored in a computer.

In digital holography, the recorded digital holograms can be reconstructed using various numerical reconstruction methods such as the angular spectrum method (ASM)^1^, Fresnel diffraction method (FDM)^35^, convolution method^36^, and so on. We used FDM in both systems (with and without Fresnel bi-prism) because it satisfies the distance criterion for propagation. ASM and convolution-based methods are exact methods that are constrained to shorter propagation distances and induce aliasing in reconstruction for large objects that require longer propagation distances. Zero padding can be used in both ASM and convolution methods to reconstruct large objects over longer distances, but it significantly increases the execution time in numerical reconstruction processing. As a result, FDM is considered the most appropriate for this work. The complex amplitude of the object recorded in digital holograms can be reconstructed using the Fresnel diffraction method, represented as

5 $$\begin{gathered} O_{o} (\xi ,\eta ) = \frac{\exp ( - jkz)}{{j\lambda z}}\exp \left( {\frac{{ - j\pi (x^{2} + y^{2} }}{\lambda z}} \right) \hfill \\ *FT\left[ {E_{h} (x,y)\exp (\frac{{ - j\pi (x^{2} + y^{2} )}}{\lambda z})} \right] \hfill \\ \end{gathered}$$

where *O*_*O*_*(ξ, η)* and *E*_*h*_*(x, y)* are complex amplitude distributions at the object plane and hologram plane, respectively. FT represents Fourier transform, *k* = 2*π/λ*, λ is the source wavelength, *z* is the propagation distance, and *j* = *√−1*. For reconstruction, two conjugate orders, mentioned in Eq. (6), are filtered out in the Fourier domain and then propagated using the FDM. This filtering removes the constant DC terms and other conjugate orders.

## Experimental procedure and results

**Figure 2:**
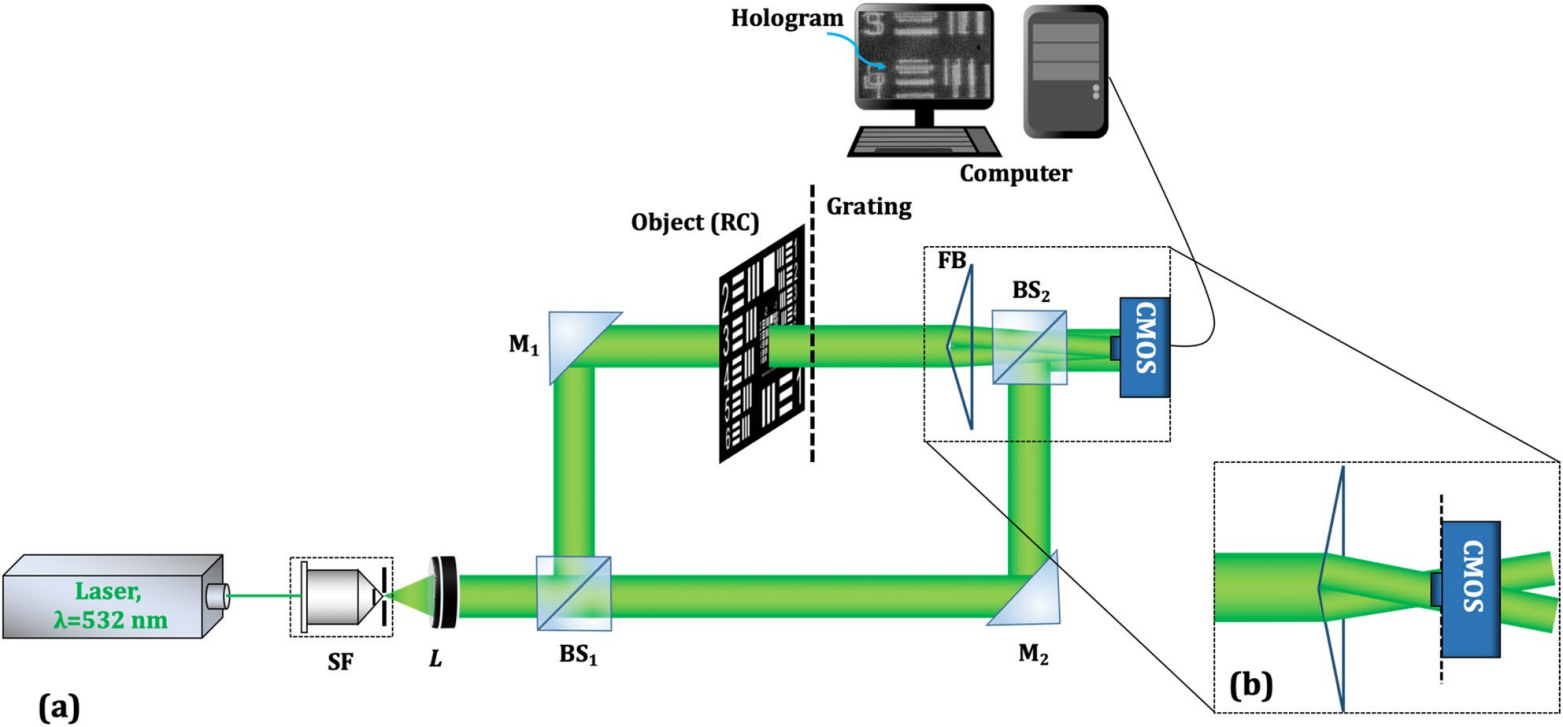
(**a**) Schematic of the experimental setup and (**b**) enlarged view of two FOV generations by FB (excluding the reference beam and BS_2_).

**Figure 3:**
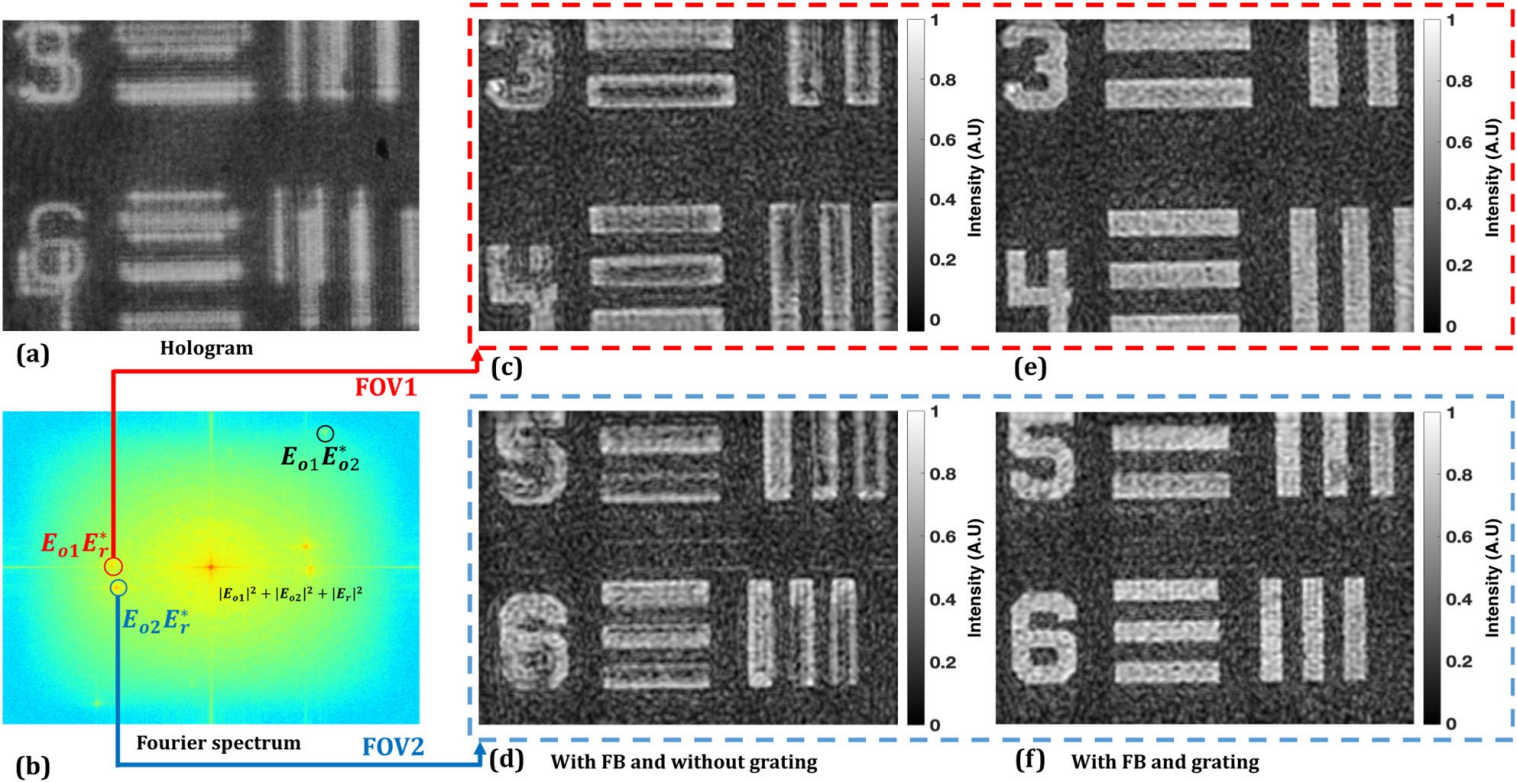
Experimental results of the proposed DH systems: (**a**) recorded multiplexed digital hologram, (**b**) Fourier spectrum of (**a**); (**c**) and (**d**) the amplitude reconstructed images corresponding to two FOVs ${(E}_{o1}{E}_{r}^{*}\mathrm{ and }{E}_{o2}{E}_{r}^{*})$; (**e**) and (**f**) are the improved reconstructed images corresponding to two FOVs when a diffraction grating is used just after the object.

Figure 2a shows the schematic of the experimental setup of the proposed DH system. A laser beam of wavelength 532 nm is expanded by the spatial filter (with 40X microscopic objective and 5 μm pinhole) and collimated by using a lens (L, *f* = 200 mm). The collimated laser beam, of diameter ~ 25 mm, is divided into the object and reference beams by using a beam splitter (BS_1_). The object beam is allowed to pass through the USAF resolution chart (RC). The key element of the proposed setup is Fresnel bi-prism (apex angle ~ 176˚, refractive index ~ 1.51, material—BK-7), which is placed after the RC, at a distance of 145 mm, and it divides the object beam into two FOVs. The image sensor (Matrix Vision, CMOS sensor, the resolution is 2592 × 1944 and 2.2 μm pixel size) is placed at the plane of superposition of the two FOVs generated by the Fresnel bi-prism. Figure 2b shows the enlarged view of two FOV generations by the Fresnel bi-prism and the position of the image sensor. The plane reference beam is allowed to interfere, with the help of BS_2_, in off-axis geometry with two FOVs of the object beam, at the active region of the image sensor to form a multiplexed digital hologram. The off-axis configuration is created by providing a small tilt to BS_2_. Figure 3a shows the recorded multiplexed digital hologram in which it is clearly seen that the multiple object information corresponding to different areas is superimposed onto the same photosensitive area of the image sensor. The whole object wave is composed of two captured partial object waves.

Since the reference beam is common for both the two FOVs of the object, each pair of beams encodes a different wavefront, and the image sensor records three separable off-axis interferences when seen in the spatial frequency domain of the multiplexed hologram: two between the reference beam and each of the FOVs and one between the two FOVs, as depicted in Fig. 3b. The intensity distribution of the interference pattern formed by the two object beams and one reference beam at the image sensor plane can be represented as

6 $$E = \left| {E_{o1} + E_{o2} + E_{r} } \right|^{2} = \left| {E_{o1} } \right|^{2} + \left| {E_{o2} } \right|^{2} + \left| {E_{r} } \right|^{2} + E_{o1} E_{o2}^{*} + E_{o2} E_{o1}^{*} + E_{o1} E_{r}^{*} + E_{o2} E_{r}^{*} + E_{r} E_{o1}^{*} + E_{r} E_{o2}^{*}$$

where, ${E}_{o1}$ and ${E}_{o2}$ are the amplitudes of the object beams corresponding to the two FOVs, ${E}_{r}$ is the electric field amplitude of the reference beam, and * represents complex conjugate. In the spatial-frequency domain (see Fig. 3b), the first three terms on the right side of Eq. (2) represent auto-correlation (AC) elements; the other six terms represent the cross-correlation (CC) between the waves.

**Figure 4:**
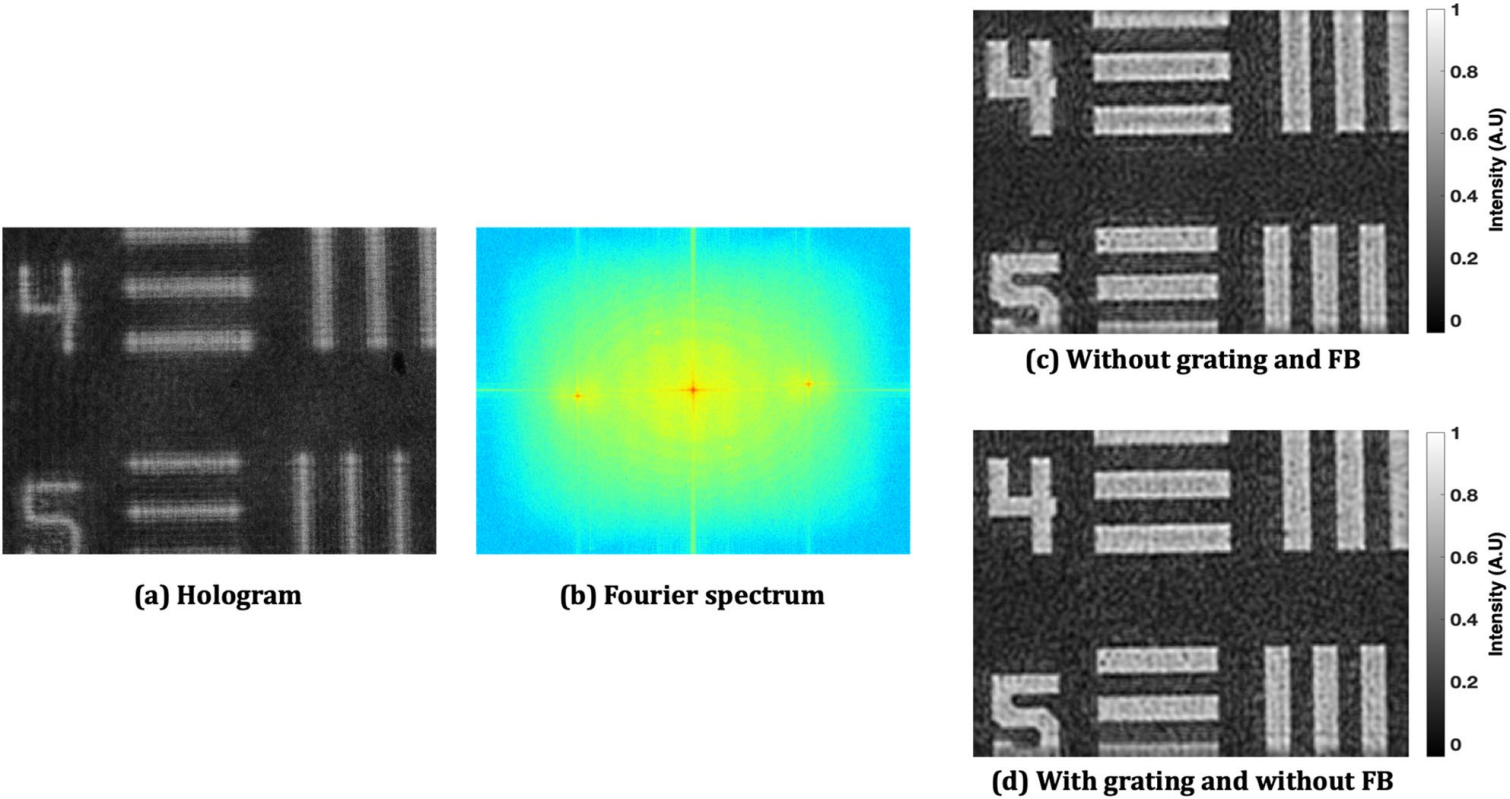
Experimental results of the Mech-Zehnder type DH system: (**a**) recorded digital hologram, (**b**) Fourier spectrum of (**a**); (**c**) the reconstructed image, and (**d**) the reconstructed image when the grating is used just after the object.

The numerical reconstruction based on the Fresnel diffraction method^36^ is carried out twice corresponding to the two FOVs by spatial filtering of ${E}_{o1}{E}_{r}^{*}\mathrm{ and }{E}_{o2}{E}_{r}^{*}$. Figure 3c,d show the amplitude reconstructed images corresponding to two FOVs ${(E}_{o1}{E}_{r}^{*}\mathrm{ and }{E}_{o2}{E}_{r}^{*})$, hence, equivalent to double the recording area, if compared with the case when no Fresnel bi-prism is used in the object beam (see Fig. 4). The experimental results of the Mech-Zehnder type DH system, i.e. without the use of Fresnel bi-prism, are presented in Fig. 4. Figure 4a shows the recorded hologram, the sensor FOV equivalent to the elements 4 and 5 of group 0 of the USAF test target. On the other hand, the proposed DH system has the ability to record and retrieve the optical FOV equivalent to elements 3, 4, 5, and 6 of the group 0. Therefore, the system shows imaging capability to obtain a double FOV.

**Figure 5:**
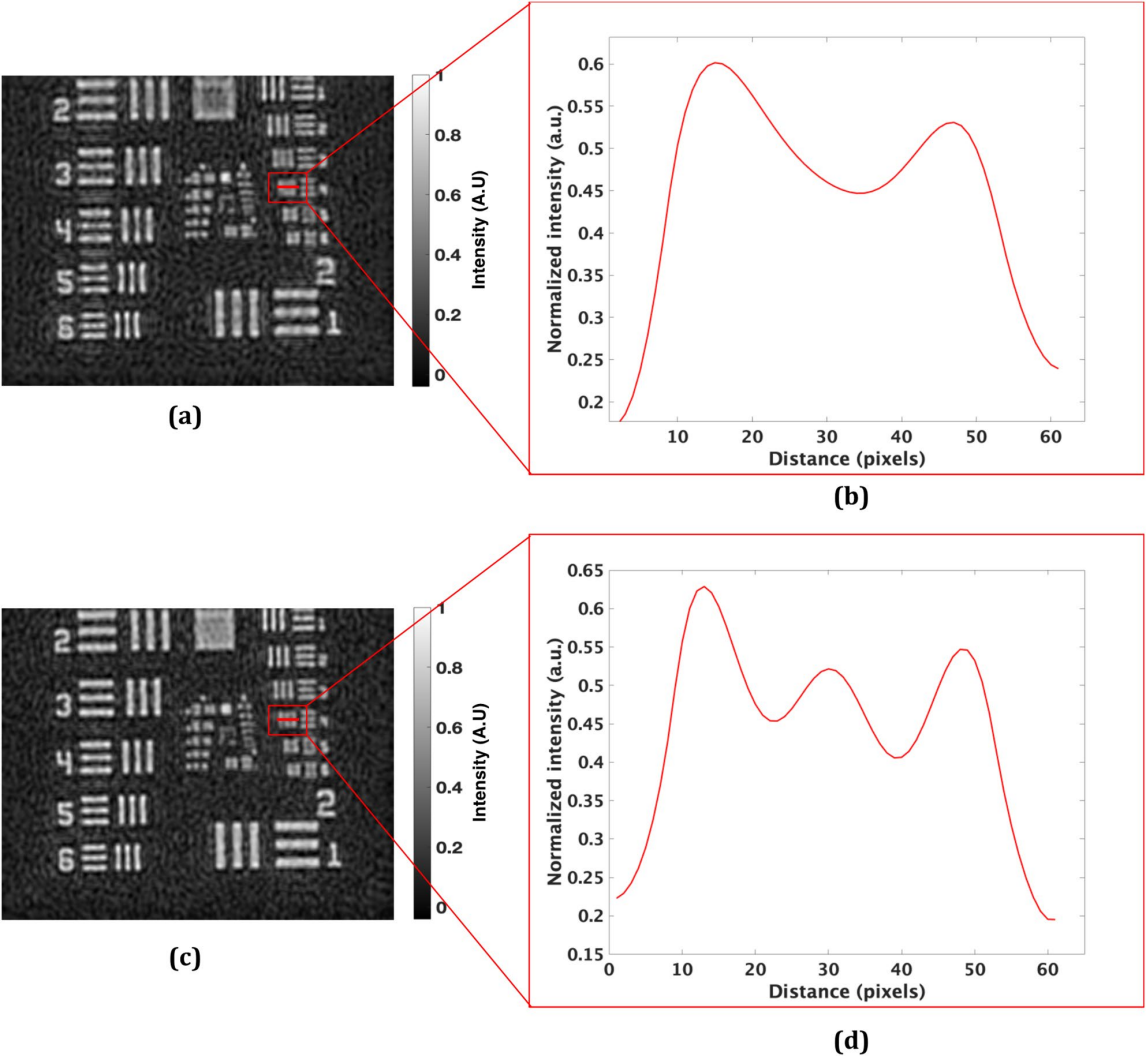
Amplitude reconstructed images of the Mech-Zehnder type DH system (**a**) without and (**c**) with grating; (**b**) and (**d**) corresponding normalized intensity profiles calculated along the red line shown in (**a**) and (**c**).

However, at the same time, it is observed (as in Fig. 3c,d) that the system loses image resolution, therefore, making it a weaker candidate for practical applications. This is due to the fact that the complex-conjugate terms (${{{E}}}_{{{o}}1}{{{E}}}_{{{r}}}^{{*}}{and}{{{E}}}_{{{o}}2}{{{E}}}_{{{r}}}^{{*}}$) corresponding to the two FOVs are closely packed and held fixed in the spatial frequency domain of the multiplexed digital hologram. The spatial frequency distributions of the complex-conjugate terms (${{{E}}}_{{{o}}1}{{{E}}}_{{{r}}}^{{*}}{ and} {{ }{{E}}}_{{{o}}2}{{{E}}}_{{{r}}}^{{*}}$) are slightly superimposed due to the fixed small refraction angle of the Fresnel bi-prism and therefore, the reconstructed images are degraded. The Fresnel bi-prism employed in this proposed experimental setup does not provide any control on varying the position of these CC terms in the spatial frequency domain for the two FOVs, by changing the orientation of the interference fringes with the same reference beam. So, to improve the resolution in the present setup, we employed a one-dimensional diffraction grating (500 lines per mm) on the object, redirecting the higher spatial-frequencies of the spectrum of the object toward the image sensor^36,37^. The grating is placed in near contact with the object, in order to avoid the cross-talk among different orders. To demonstrate proof of concept, we conducted an experiment with and without the diffraction grating. The experimentally obtained results are depicted in Fig. 5. Figure 5a shows the reconstructed image obtained without the use of grating and 5(b) shows the intensity profile across the red line of case 5(a) in the red square (group 1 element 4). On the other hand, an image with the improved resolution is retrieved when the grating is placed on the object, as depicted in Fig. 5c. Figure 5d shows the intensity profile across the red line. Comparing the profile in Fig. 5d with Fig. 5b, the resolution is effectively improved. The experimental results, by employing the diffraction grating in the proposed DH system for FOV extension, are presented in Fig. 3e,f. The reconstruction results of Fig. 3e,f, in contrast to Fig. 3c,d, give clear evidence of optical resolution improvement in the proposed FOV extension DH system based on Fresnel bi-prism.

**Figure 6:**
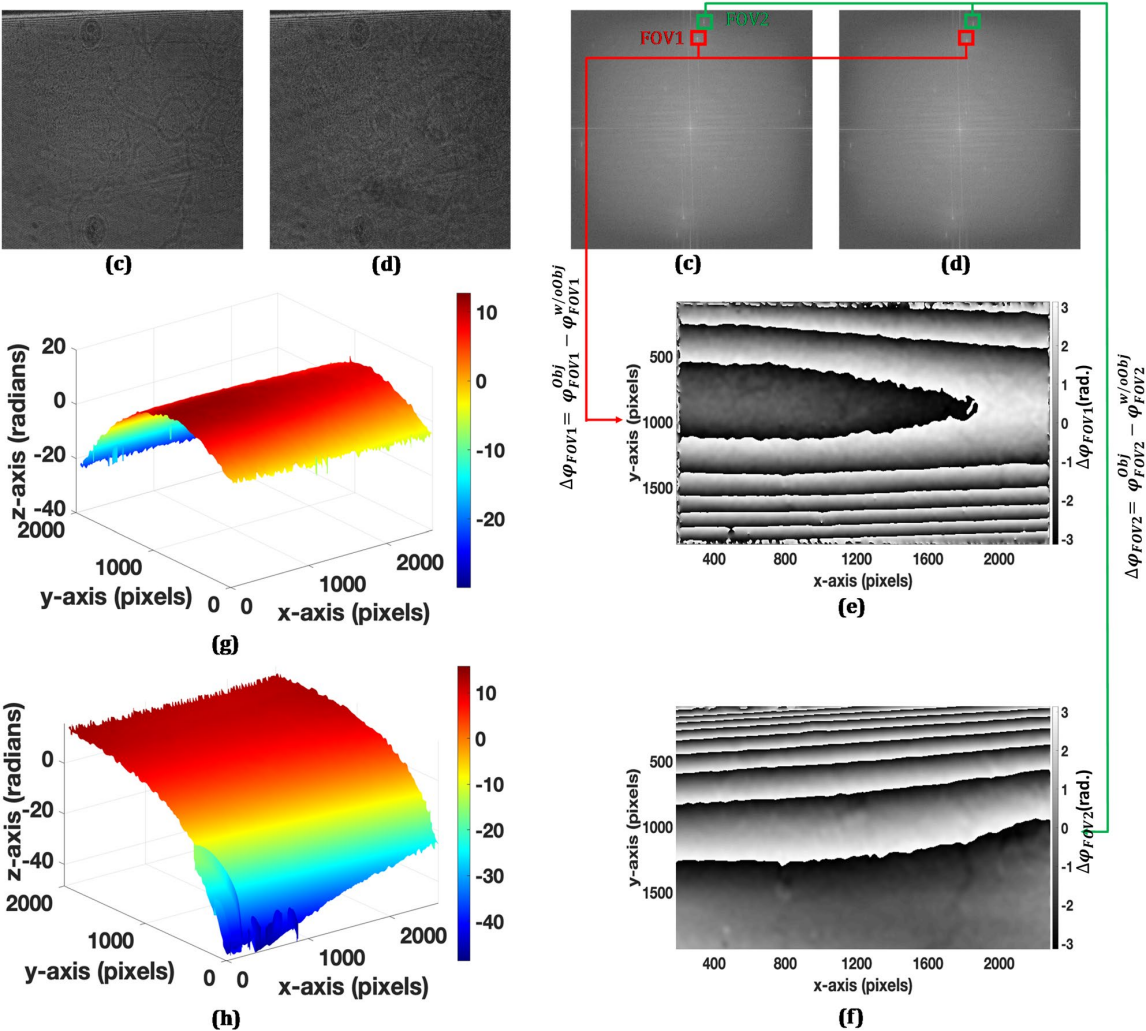
(**a**) Recorded digital hologram with the glass plate, (**b**) recorded digital hologram without the glass plate, (**c**–**d**) Fourier transform spectrums of (**a**) and (**b**), (**e**–**f**) wrapped phase difference maps corresponding to FOV1 and FOV2, respectively, and (**g**–**h**) unwrapped phase distributions corresponding to (**e**–**f**), respectively.

**Figure 7:**
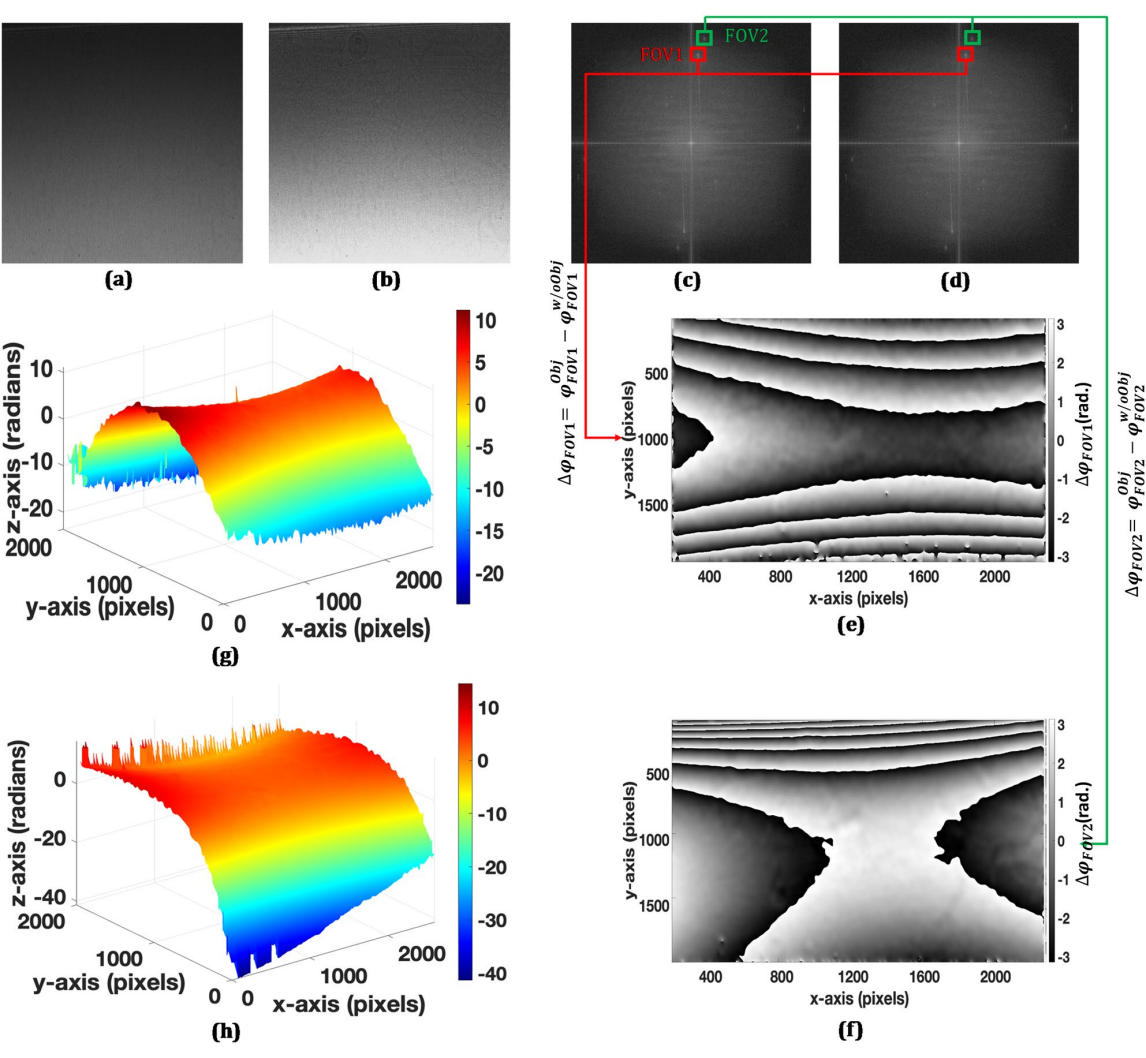
(**a**) Recorded digital hologram with the candle flame, (**b**) recorded digital hologram without the glass plate, (**c**–**d**) Fourier transform spectrums of (**a**) and (**b**), (**e**–**f**) wrapped phase difference maps corresponding to FOV1 and FOV2, respectively, and (**g**–**h**) unwrapped phase distributions corresponding to (**e**–**f**), respectively.

Holographic methods have been demonstrated to be versatile tools for the solution of many non-destructive measurement problems. Due to the potential capability of the proposed DH system to retrieve phase information of a large area (as double of its counterparts), it can be used as a non-destructive, optical measurement and inspection tool in a wide range of applications. We experimentally demonstrate some of its optical metrological applications by extracting the phase information of the objects such as an optical glass plate (23 mm × 22 mm × 1.5 mm) and candle flame. In this experiment, two holograms: one in the presence of the object (glass plate or candle flame) and another without the object, are recorded by the proposed DH system. These objects are placed at the position of the resolution chart in Fig. 2a. Figure 6a–b show the recorded holograms with and without the glass plate, respectively. The Fourier spectrums of these two holograms are depicted in Fig. 6c–d, where ± 1 orders corresponding to two FOVs are clearly seen. The phase distribution of the object wavefronts for the two holograms and for both the FOVs, are numerically reconstructed separately from these recorded digital holograms by the Fresnel diffraction method^1,35^. The interference phase, i.e., the phase difference between two states of the object (with and without object), is calculated directly by modulo 2π subtraction^38,39^. Figure 6e–f show the wrapped phase difference maps corresponding to the two FOVs. The numerically calculated interference phase remains wrapped in the range (− π, + π) radian and may range over an interval greater than 2π. This 2π phase discontinuity was corrected by the PUMA phase unwrapping method^40^ to obtain a continuous unwrapped phase. The unwrapped phase distributions are aberration correlated. The aberration correlation can be accomplished either by numerical methods or physical methods. The numerical methods are implemented by quantifying and removing aberrations during the digital reconstruction process, whereas the physical methods include, for example, subtraction of the phase of a reference hologram without the object from the phase of the object hologram. Here, we used the physical method, i.e. the object phase is subtracted from the reference hologram phase to obtain aberration-free phase distribution. Figure 6g,h show the obtained unwrapped phase distributions corresponding to the wrapped phase maps of Fig. 6e,f, respectively. The obtained continuous unwrapped phase distributions can further be used for measuring various physical parameters of the object under study including the deformation, displacement, strain/stress, vibration, refractive index, density, temperature, etc.^41–45^. Similarly, the recorded digital holograms, their Fourier spectrums, and phase distribution information, corresponding to two FOVs of a candle flame, are depicted in Fig. 7. The obtained phase distribution results corroborate the feasibility of the proposed DH system and indicate its potential applications for the non-destructive testing, shape and dynamic deformation measurements, and experimental stress analysis with the additional advantage of double the camera FOV.

## Conclusion

In summary, we proposed and experimentally demonstrated a new configuration of a single-shot multiplexed DH system based on Fresnel bi-prism. We have shown that it extends the FOV by recording two different areas of the object beam, contrary to a fraction of it, i.e. sensor FOV, as in the case of the optical DH system. However, due to overlapping of the spatial frequency distributions of the complex-conjugate terms in the frequency domain, the resolution of the reconstructed images is severely degraded. The resolution is kept at almost the same level as that of an optical DH system by employing a diffraction grating in near contact with the object which allows recording the high spatial frequencies and optimization of the information capacity. The feasibility of the proposed system is experimentally demonstrated by imaging and numerical reconstruction of two different areas of the USAF resolution test target, and analysis of the scientific and industrial measurement applications by calculating the phase distribution of a glass plate and a candle flame. The proposed system has prospective applications in multiplexing microscopy, quantitative phase imaging, high-speed imaging, 3D imaging applications, and optical metrology.

## Data Availability

Data underlying the results presented in this paper are not publicly available at this time but may be obtained from the authors upon reasonable request.
